# The Mammalian Diving Response: Inroads to Its Neural Control

**DOI:** 10.3389/fnins.2020.00524

**Published:** 2020-06-05

**Authors:** W. Michael Panneton, Qi Gan

**Affiliations:** ^1^Department of Pharmacological and Physiological Science, School of Medicine, Saint Louis University, St. Louis, MO, United States; ^2^Department of Pediatrics, School of Medicine, Saint Louis University, St. Louis, MO, United States

**Keywords:** marine mammals, respiratory chemoreceptors, bradycardia, medulla, SIDS, headache, stroke, arrhythmias

## Abstract

The mammalian diving response (DR) is a remarkable behavior that was first formally studied by Laurence Irving and Per Scholander in the late 1930s. The DR is called such because it is most prominent in marine mammals such as seals, whales, and dolphins, but nevertheless is found in all mammals studied. It consists generally of breathing cessation (apnea), a dramatic slowing of heart rate (bradycardia), and an increase in peripheral vasoconstriction. The DR is thought to conserve vital oxygen stores and thus maintain life by directing perfusion to the two organs most essential for life—the heart and the brain. The DR is important, not only for its dramatic power over autonomic function, but also because it alters normal homeostatic reflexes such as the baroreceptor reflex and respiratory chemoreceptor reflex. The neurons driving the reflex circuits for the DR are contained within the medulla and spinal cord since the response remains after the brainstem transection at the pontomedullary junction. Neuroanatomical and physiological data suggesting brainstem areas important for the apnea, bradycardia, and peripheral vasoconstriction induced by underwater submersion are reviewed. Defining the brainstem circuit for the DR may open broad avenues for understanding the mechanisms of suprabulbar control of autonomic function in general, as well as implicate its role in some clinical states. Knowledge of the proposed diving circuit should facilitate studies on elite human divers performing breath-holding dives as well as investigations on sudden infant death syndrome (SIDS), stroke, migraine headache, and arrhythmias. We have speculated that the DR is the most powerful autonomic reflex known.

## Introduction

The complexity of an animal’s behavior is paralleled by the complexity of the neural systems driving that behavior. Indeed, numerous neurons within the mammalian brain modulate autonomic activity and these areas are interconnected in complex ways. Despite this complexity, an orderly functional organization exists because specific autonomic responses result from a specific stimulus, and these adjustments are appropriate to physiological needs. Moreover, behaviors that serve basic vegetative functions are usually less complex and more uniform across species. The substrate for “simple” reflexive behaviors is thought to be neural circuits located within the brainstem and spinal cord; it is probable that some of these same circuits are influenced by the more rostral parts of the brain and are utilized in more complex behaviors. It is therefore worthwhile to direct considerable effort toward studying those circuits that are the simplest, the most organized, and the most automatic. A behavior validating such a statement is the mammalian diving response (DR), a mechanism that operates in a variety of animals across a wide range of circumstances, thus suggesting it to be of fundamental physiological significance (Scholander, 1963). The threat of asphyxiation in numerous species quite distantly related was the common theme in the pioneering studies of Irving and Scholander, who showed an organism’s primary response was bradycardia, i.e., the dive reflex. A history of their work on various species’ responses to asphyxia has recently been documented (Hagen, 2018). The somatoautonomic DR is powerful and modulates intrinsic rhythms such as respiration and heart rate, as well as basic homeostatic reflexes such as the chemoreceptor and baroreceptor reflexes. The neural pathways mediating the DR are being explored; this treatise provides a summation of current understanding.

The mammalian DR is usually considered to consist of three independent reflex behaviors: an apnea, a parasympathetically mediated bradycardia and a sympathetically mediated peripheral vasoconstriction (Irving, 1938, 1939; Irving et al., 1942); splenic contraction is sometimes considered a fourth behavior by some (Cabanac et al., 1997, 1998; Cabanac, 2000). These autonomic adjustments are marked mostly in marine mammals such as seals, dolphins, or whales, which spend considerable time submerged underwater. Thus, when pinnipeds or cetaceans dive underwater, oxygen from air becomes non-existent, and the animal is forced to use oxygen bound either to hemoglobin in its blood or myoglobin in its muscles (Guyton et al., 1995; Noren and Williams, 2000), or to depend on anaerobic glycolysis (see Panneton, 2013 for discussion and references). Many of the physiological consequences of diving have been deciphered, and adaptations of cetaceans and pinnipeds widely reported (Kooyman et al., 1981; Butler and Jones, 1982, 1997; Blix and Folkow, 1983; Elsner and Gooden, 1983; de Burgh Daly, 1984; Kooyman and Ponganis, 1998; Panneton, 2013; Davis, 2014). The DR is also found in birds (prominently in ducks and penguins) (Butler and Jones, 1982, 1997; Kooyman and Ponganis, 1998; Ponganis and Kooyman, 2000), and even fish show a bradycardia in hypoxic environments (Scholander, 1963; Elsner and Gooden, 1983; Farrell, 2007).

This review focuses on the *neural* control of the DR only in terrestrial mammals, particularly rodents, and differs from most previous reviews which emphasize the adaptations and physiological consequences of aquatic mammals to underwater submersion with little or no discussion on central neural integration. This differs from a previous review (Panneton, 2013) by emphasizing a brainstem reflex circuit driving the DR and a more detailed exploration on its suprabulbar control as well as how this response may help humans clinically. Our premise is to decipher a conserved neural circuit driving the DR, which we suspect is uniform across species. We do this in rodents simply because these small mammals are abundant and bred for laboratory use, and much is known of their physiology and nervous systems (NSs), and rodents are significantly less challenging ethically than use of large marine mammals. Neural circuits located within the brainstem and the spinal cord are described, since they are the simplest, most organized, and most automatic. If our hypothesis is correct, the circuit outlined for rodents should be mimicked in the brains of higher marine mammals, as well as be applicable to humans. It may also provide a base for future studies on the mechanisms underlying the stunning physiologic changes induced in the mammalian DR. This review merely augments the wealth of knowledge obtained from the numerous studies obtained from marine mammals.

**FIGURE 1:**
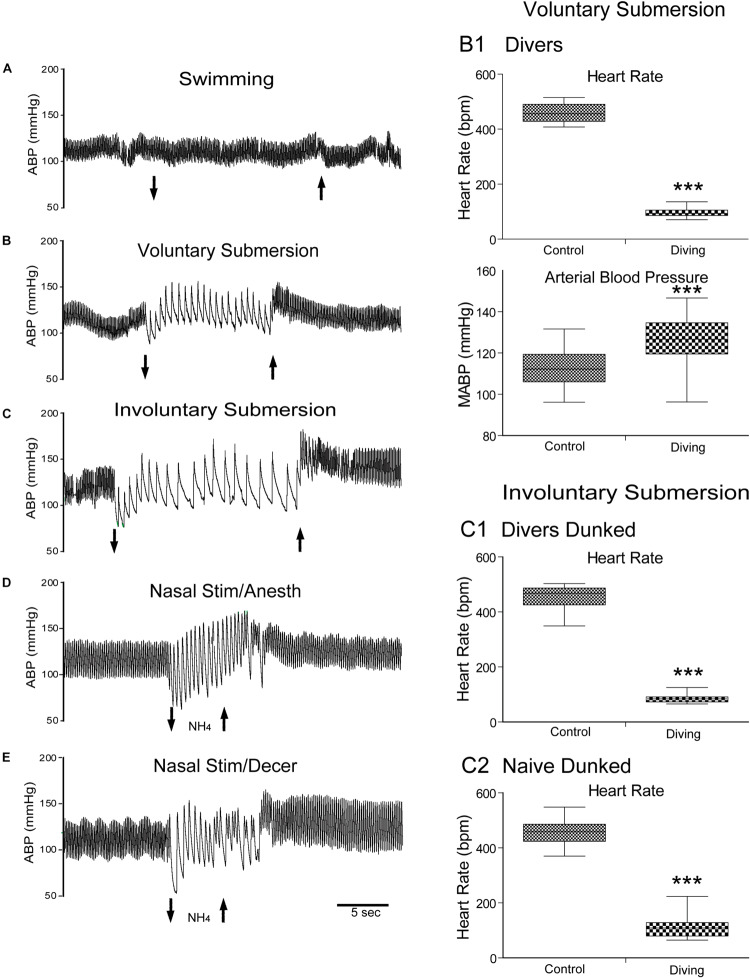
The mammalian diving response can be induced in different preparations of laboratory rodents. Cardiovascular responses either to swimming, underwater submersion, or nasal stimulation are shown. Traces of arterial blood pressure (ABP) of rats swimming **(A)**, voluntarily diving underwater **(B)**, involuntarily dunked underwater **(C)**, stimulated nasally with ammonia vapors under anesthesia **(D)**, and stimulated nasally with ammonia vapors after decerebration **(E)** are presented. Note the marked bradycardia and increase in ABP after submersion **(B,C)** or nasal stimulation **(D,E)** but no changes after swimming on the water’s surface **(A)**. Composite of cardiovascular results of rats voluntarily diving underwater (**B1**; 30 rats, *N* = 104), involuntary dunking of rats trained to dive underwater (**C1**; 17 rats, *n* = 59), and involuntary dunking of untrained rats naïve to water (C2; 21 rats, n = 39). These charts show that changes in the heart rate (HR) and mean ABP (MABP) were highly significant after voluntary submersion **(B1)**, and HR after involuntary submersion of trained rats **(C1)** or untrained naïve rats **(C2)**. Compare the bradycardic responses to underwater submersion in these charts and note the wider spread in naïve rats than those familiar to water, suggesting more stress in this group as well as potential suprabulbar modulation. ***P < 0.001; paired samples *T*-test for **C1**–**C3**. **A**–**E** are reprinted from J. Appl. Physiol., 108, Panneton et al., The rat: a laboratory model for studies of the diving response, 811–820 (2010), with permission. See Panneton et al. (2010b) for discussion on these different preparations.

The DR is found in all mammals studied, including those terrestrial (Butler and Jones, 1982, 1997; Blix and Folkow, 1983; Elsner and Gooden, 1983; de Burgh Daly, 1984; Ferretti, 2001; Davis et al., 2004; Foster and Sheel, 2005). An animal model for many years was the feral muskrat, *Ondatra zibethicus* (Martin et al., 1977; Doyle et al., 1988; Panneton, 1990, 1991a,b; Panneton and Watson, 1991; Panneton and Yavari, 1995; Panneton et al., 1996, 2000; McCulloch and Panneton, 1997; McCulloch et al., 1999a) since this semi-aquatic rodent possesses a brisk and reliable DR even when anesthetized (Koppányi et al., 1929; Irving, 1939; Drummond and Jones, 1979; McCulloch and Jones, 1990). However, this feral species which must be trapped wild is somewhat difficult to obtain reliably, and no information about its brain was known; for these reasons, we sought another animal model. We found the common laboratory rat is better suited for deciphering the neural circuits driving the DR. Rats can be trained easily to dive underwater (McCulloch, 2012; Panneton et al., 2014; Supplementary Video S1), and the DR can be documented (Figure 1) with implanted telemetric transmitters (McCulloch et al., 2010; Panneton et al., 2010a, b, 2012a, 2014). The DR has also been documented recently in mice (Hult et al., 2019); the authors also provide a simple training protocol for these irascible creatures. These studies have shown that responses in diving rodents mimic those of marine mammals; the use of laboratory rodents for studying the DR has been reviewed recently (McCulloch, 2012). Thus, we and others (Lin, 1974; Lin and Baker, 1975; McCulloch et al., 1997, 2010; McCulloch, 2005; Fahlman et al., 2011) have shown that laboratory rats have an innate DR marked by a bradycardia reaching 80% in 100% of rats 100% of the time during submersion (Figure 1), which is typical of reflex behaviors. This reproducibility implies that reflex circuits are probably utilized by mammals to manifest the DR. However, the central neural pathways driving the DR have been relatively unexplored in any species. A purpose of our laboratory is to define the neural circuits driving the DR, especially those inducing the apnea, bradycardia, and peripheral vasoconstriction. We consider the DR the most powerful autonomic reflex known.

It is known that the apnea (breath-hold) is maintained during the DR despite gross alterations of blood gases, which reach levels that would normally drive respiration. Thus, it has been suggested that respiratory chemoreceptors, which normally induce vigorous ventilation when activated, are inhibited (Elsner et al., 1977; de Burgh Daly, 1984; McCulloch and West, 1992; McCulloch et al., 1997); this is indeed the case (Panneton et al., 2010a). Second, there is a dramatic bradycardia mediated by the vagus nerve of the parasympathetic NS, which reduces cardiac output. Third, there is vasoconstriction in the vascular beds to non-essential organs (i.e., muscle, abdominal organs, skin) while the two most essential organs, the heart and the brain, remain perfused. The vasoconstriction seen with diving is mediated via the sympathetic NS (Yavari et al., 1996; McCulloch et al., 1999b), maintaining internal oxygen stores for the brain and the heart (Ollenberger and West, 1998b).

## Experimental Procedures

Discussions of techniques employed will not be emphasized in this review. However, interested readers are referred to manuscripts with numerous references detailing the location of the anterior ethmoidal nerve (AEN; Panneton, 1991a) and its recording/stimulation (McCulloch et al., 1999a) stimulating the nasal mucosa with vapors (Panneton, 1991b; Panneton and Yavari, 1995; Yavari et al., 1996; Panneton et al., 2010b), for transganglionic transport techniques from primary afferent fibers (Panneton, 1991a; Panneton et al., 2006; Panneton and Gan, 2014), for pharmacological blockade of central pathways (Panneton and Yavari, 1995; McCulloch et al., 1999b), for neuroanatomical tract-tracing of central pathways (Panneton et al., 2000, 2006), for the use of cFos as a neuroanatomical marker of function (Panneton et al., 2010a, 2012a), for the training of rats and mice to dive (McCulloch, 2014; Panneton et al., 2014; Hult et al., 2019), and for deployment of telemetric transmitters to measure cardiovascular changes in both trained rats (Panneton et al., 2010a, b, 2012a) and mice (Hult et al., 2019). The limitations of these techniques are discussed in these manuscripts. Also, nomenclature of the brainstem, particularly the reticular formation, and the terms used by us in this review, have been defined previously (Sun and Panneton, 2005; Panneton et al., 2006).

## The Diving Response as a Reflex

**FIGURE 2:**
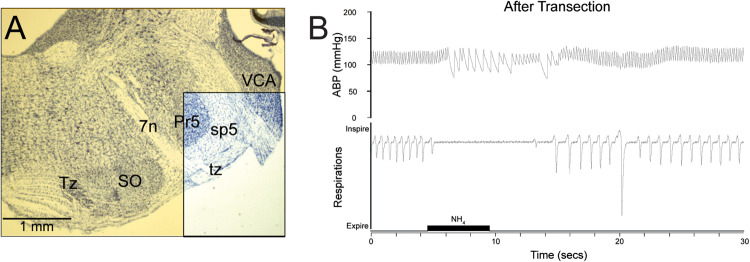
The reflex circuit driving the diving response is contained within the medulla and spinal cord. Transections through the pontomedullary junction (**A**, yellow transparency) were made, sparing only neuropil in the ventral part of the spinal trigeminal complex (**A**, boxed area), including primary afferent fibers of the AEN descending in the spinal trigeminal tract (sp5). Despite this trauma, cardiorespiratory depression similar to that seen in underwater submersion was maintained after nasal stimulation **(B)**, promoting the idea that the neural circuits driving the DR are situated in the medulla, and thus are similar to other brainstem reflex circuits. Figures are reprinted from Respir. Physiol. Neurobiol., 180, Panneton et al., Persistence of the nasotrigeminal reflex after pontomedullary transection, 230–236 (2012), with permission. See Panneton et al. (2012b) for more details.

A reflex is “an involuntary reaction in response to a stimulus applied to the periphery and transmitted to the nervous centers in the brain or spinal cord,” by definition. Most data suggest that the DR consists of three independent reflexes regulating respiration (an apneic reflex), heart rate (a bradycardic reflex), and arterial blood pressure (ABP; a vasoconstrictor reflex), respectively, but splenic contraction has also been documented in numerous species. Pharmacologic studies using peripheral application of antagonists/agonists show that heart rate and blood pressure responses can be blocked selectively while preserving the other two reflexes (Yavari et al., 1996; Elliott et al., 2002). However, peripheral autonomic fibers apparently mediate neither dive time nor surface intervals in seals after receptor blockade (Elliott et al., 2002). Moreover, similar blocking studies show that bradycardia to underwater submersion is cholinergically mediated, while sympathetic innervation is far less important (Elliott et al., 2002). The responses after stimulating the nasal mucosa with noxious vapors are similar to those of underwater submersion (Angell James and de Burgh Daly, 1969, 1972, 1973; White et al., 1974; Peterson et al., 1983; Panneton, 1990; Nakamura and Hayashida, 1992; Gieroba et al., 1994; Panneton and Yavari, 1995; Yavari et al., 1996; Kobayashi et al., 1999; Ho and Kou, 2000; Dutschmann and Paton, 2002), and both behaviors are considered reflexes by us. The circuitry for the DR is intrinsic to the medulla and the spinal cord (Panneton et al., 2012b), since the responses remain to nasal stimulation despite brainstem transection at the pontomedullary junction (Figure 2), sparing only the ventral third of the trigeminal sensory complex (Panneton et al., 2012b) and promoting its definition as a reflex. Thus, our conclusion differs from that of others (Dutschmann and Herbert, 1996, 1997, 1998a, 1999; Chamberlin and Saper, 1998; Dutschmann et al., 1998, 2004; Radulovacki et al., 2003, 2004; Chamberlin, 2004; Topchiy et al., 2009) who suggest that autonomic changes induced by trigeminal stimulation are dependent on neurons in the pons, including the parabrachial-Kölliker-Fuse nuclei and the intertrigeminal region. Maintaining a DR following pontomedullary transection implies that this life-saving response may be organized in redundant circuits, and failure or blockade of the pontine loops are compensated by medullary circuits. This is likely to happen with failure of critical NMDA receptors in the pons during progression of uncompensated hypoxia (e.g., during drowning). Since the pons receives significant ascending inputs from the trigeminal sensory relays (Panneton et al., 1994, 2006), it is reasonable to assume that the pons is an integral part of the neural circuit that mediates the DR under intact conditions. However, since all descending/ascending fibers from/to suprabulbar structures were cut yet the response was maintained, we find that these suprabulbar areas *modulate* a basic medullary reflex circuitry.

## The Stimulus

The independent reflexes comprising the DR act harmoniously toward preserving vital oxygen stores and are initiated by activating peripheral receptors. Early studies (Koppányi et al., 1929; Irving, 1939; Irving et al., 1942; Tchobroutsky et al., 1969; Dykes, 1974; Lin, 1974; Whishaw and Schallert, 1977; Gandevia et al., 1978; Drummond and Jones, 1979; Schagatay and Van Kampen, 1995) noted that submersion or wetting of nasal areas was important to induce the DR, and this has been confirmed by others numerous times. Thus, underwater submergence is the usual stimulus to induce the DR in awake animals. This spurred many investigators to perform “forced” submersions, where the animals were tethered on boards or placed in cages and dunked underwater (Koppányi et al., 1929; Scholander, 1963; Dykes, 1974; Lin, 1974; Lin and Baker, 1975; Martner et al., 1977; Drummond and Jones, 1979; Jones et al., 1982; Schagatay and Van Kampen, 1995; Panneton et al., 2010a, b). However, forcing the animals underwater usually hinders the formidable interventions necessary to monitor respiration, arterial pressure, and heart rate, as well as access to structures in the brain.

Water flowing over the nasal mucosa has been used as a stimulus (Angell James and de Burgh Daly, 1972; Gandevia et al., 1978; Drummond and Jones, 1979; Doyle et al., 1988), but high flow rates often strip mucosa from nasal bones and create blood clots. Irritating vapors (smoke, ammonia, formaldehyde) wafted over the nasal mucosa prevents gross mucosal disruption and offers better control (time and intensity) of the stimulus to induce autonomic responses similar to diving (McRitchie and White, 1974; White et al., 1974, 1975; Drummond and Jones, 1979; Doyle et al., 1988; Lee et al., 1990; Panneton, 1990, 1991b; Nakamura and Hayashida, 1992; Gieroba et al., 1994; Panneton and Yavari, 1995; Yavari et al., 1996; McCulloch and Panneton, 1997; McCulloch et al., 1999b; Ho and Kou, 2000; Ho and Kou, 2002). Finally, the electrical stimulation of the AEN also induces an apnea, bradycardia, and sympathoactivation typical of the DR (Dutschmann and Herbert, 1996, 1997, 1998a,b; McCulloch et al., 1999a; Dutschmann and Paton, 2002), but with the caveat that electrical stimulation potentially activates a wide variety of fibers, including those nociceptive and others important for sneezing, and as such may muddle interpretations of those investigating the DR.

## Nasal and Paranasal Receptive Fields

Since the mammalian DR can be induced with only snout immersion, this suggests that primary afferent fibers innervating nasal and paranasal areas may be important. Indeed, covering paranasal areas with petroleum jelly or numbing these areas with anesthetic eliminates the autonomic responses induced by submersion (Dykes, 1974; Drummond and Jones, 1979). Paranasal areas are innervated by branches of the maxillary branch of the trigeminal including its large infraorbital nerve, which innervates the ala of the nose and the upper lip, as well as the AEN of the ophthalmic division of the trigeminal, which innervates mucosa of the lateral and septal nasal walls in humans, the skin of the ala, and the vestibule and apex of the nose (Williams and Warwick, 1980; Wallois et al., 1991). The nasal mucosa has both respiratory and olfactory segments (Ross et al., 1995), but the olfactory epithelium is not considered important for the DR, since the DR remains after olfactory bulb ablation (Angell James and de Burgh Daly, 1972; McRitchie and White, 1974; Drummond and Jones, 1979; Panneton, 1990; Gieroba et al., 1994; Kratschmer, 2001). Indeed, cetaceans with their blowholes have neither olfactory bulbs nor an olfactory system, while baleen whales retain only small rudimentary olfactory organs, despite their diving prowess.

Innervation of the nasal mucosa is via free nerve endings from small diameter fibers (Cauna et al., 1969), most of which are C-fibers and contain peptides, notably calcitonin gene-related peptide (CGRP) and substance P (Petersson et al., 1989; Silverman and Kruger, 1989; Stjärne et al., 1989; Finger et al., 1990; Silver et al., 1991; Spit et al., 1993; Matsuda et al., 1994, 1998), derived from trigeminal ganglion neurons (Silverman and Kruger, 1989; Ichikawa et al., 1993; Matsuda et al., 1994; Schaefer et al., 2002). Most of these fibers are sensory in function, and many respond as chemoreceptors, creating the “common chemical sense,” or chemethesis (Cain and Murphy, 1980; Green and Lawless, 1991; Viana, 2011; Green, 2012) since sensations, including pain, can be elicited from stimulating the human nasal mucosa (Handwerker and Kobal, 1993; Thürauf et al., 1993; Cometto-Muñiz and Cain, 1997; Cometto-Muniz et al., 1998, 2001; Hummel et al., 2003). While inhaled irritants may stimulate these small free nerve endings directly, “solitary chemoreceptive cells” (SCCs) (Finger et al., 2003; Tizzano and Finger, 2013) within the mucosa of the upper respiratory tract of amniotes, including the nasal mucosa of humans (Barham et al., 2013), may also serve as intermediaries in a nociceptive or chemosensor pathway. SCCs in the nasal mucosa are found mostly anteriorly and innervated by small polymodal nociceptors of the trigeminal nerve (Finger et al., 2003; Tizzano et al., 2010). When these SCCs are activated they induce respiratory reflexes including apnea (Tizzano et al., 2010; Tizzano and Finger, 2013); their peripheral distribution greatly overlaps that of AEN innervation.

## The Sensory Nerve

We consider the AEN as the “gatekeeper” nerve since it is the first to sense noxious gases or water entering the nasal passages. Stimulating the peripheral receptors of the AEN would prevent such toxins from entering the upper respiratory passages by inducing an apnea. While there are several reports in the literature documenting nerves innervating the blowholes of cetaceans, only motor fibers from the facial nerve are described; the sensory nerves from the trigeminal were rarely, if ever, considered. We suspect, however, that a nerve analogous to the AEN exists in marine mammals, and this nerve also functions as a gatekeeper of their respiratory system. More research may prove this to be the case.

The AEN of terrestrial animals contains both mechanoreceptors and chemoreceptors (Silver et al., 1986; Lucier and Egizii, 1989; Wallois et al., 1991; Sekizawa and Tsubone, 1994, 1996; Sekizawa et al., 1996, 1998; McKeegan et al., 2002) responsive to a variety of stimuli. Most of its fibers are small diameter in the Aγ or C range (Beidenbach et al., 1975; McCulloch et al., 1999a) and reach between the mucosal epithelial cells toward tight junctions (Cauna et al., 1969; Finger et al., 1990; Spit et al., 1993). The central fibers of the AEN descend in the ventral third of the spinal trigeminal tract (Panneton, 1991a; Panneton et al., 2006) and send fibers into the trigeminal sensory complex and lateral reticular formation. Moreover, CGRP in the lateral reticular formation, a peptide contained in numerous fibers of the AEN, is lost after unilateral trigeminal rhizotomy (Panneton and Gan, 2014), suggesting a direct route for primary afferent fibers to modulate cardiac and vascular activity during underwater submersion. The infraorbital nerve is very large in rodents and has numerous fibers responsive to multiple stimuli. It also sends central fibers in the spinal trigeminal tract and all trigeminal sensory nuclei (Panneton et al., 2010c, 2017), but its trigeminal distribution is much wider than that of the AEN.

Nevertheless, acute transection of the AEN attenuates the apnea and ABP changes, and greatly attenuates the bradycardia to nasal stimulation (Rybka and McCulloch, 2006), but such transection does not impair the induction of the DR in voluntary diving rats or nasally stimulated rats when allowed to survive for several days after transection (Chotiyanonta et al., 2013; McCulloch et al., 2016; McCulloch and DiNovo, 2018). Interestingly, the transection of the AEN never attenuated the rise in arterial pressure induced by the nasopharyngeal stimulation, possibly since posterior parts of the nasal mucosa are innervated by other nerves.

The AEN innervates only the nares partially, as well as anterior parts of the nasal mucosa, thus it is the first sensor to assess incoming air and earns its moniker as gatekeeper. However, the posterior aspects of the nasal mucosa receive several nerves branching from the maxillary division of the trigeminal, and these still were intact in these preparations. These small nerves to the posterior mucosa indeed effect cardiorespiratory reflexes induced by stimulation of the nasal mucosa (Kanamaru et al., 1999, 2001; Mutoh et al., 2000, 2001). The central termination of fibers of some posterior nasal nerves has been demonstrated (McCulloch et al., 2018), and they maintain the somatotopy dictated for the medullary dorsal horn (MDH; Panneton et al., 2017). Other paranasal nerves/areas also align to somatotopy in the MDH (McCulloch et al., 2018); all converge on central terminal fields related to the nose. It is of interest that tracers transported transganglionically after large injections into the infraorbital nerve (Panneton et al., 2010c) labeled the misplaced substantia gelatinosa just dorsal to that labeled from the AEN (Panneton, 2013; Panneton et al., 2017), again conforming to appropriate somatotopy.

These discussions on the innervation of the nasal mucosa must be considered moot, however, since water does not flow through the nose of voluntarily diving rats or mice, and certainly not in marine species without nasal cavities, but may be more important to consider in rats nasally stimulated with obnoxious vapors or water. Chotiyanonta et al. (2013); McCulloch et al. (2016), and McCulloch and DiNovo (2018) provide lengthy discussions on the retention of the DRs after the AEN section, speculating that sprouting of retained central fibers reinnervate denervated areas of the MDH. Another possibility is that growth of nearby non-lesioned peripheral fibers from nerves innervating areas of the nares may compensate for the loss of AEN fibers. Nevertheless, McCulloch et al. (2018) show overlap of central projections from areas surrounding the nares. If a diving rat does not flow water over its nasal mucosa during underwater submersion to induce the physiological manifestations we call the mammalian DR, perhaps the innervation of the initial portal to the upper respiratory tract, the nares, is most important. This is supported by those (Dykes, 1974; Drummond and Jones, 1979) who initially injected local anesthetic in paranasal areas and inhibited the DR.

## The First Synapse

The sensory stimulus is linked to motor output via a reflex arc, “a route followed by nerve impulses in the production of a reflex act, from the peripheral receptor organ through the afferent nerve to the central NS synapse and then through the efferent nerve to the effector organ.” Peripheral physiologists know the stimulus (underwater submersion) as well as the output (e.g., an apnea via central inhibition of respiration, bradycardia via the vagus nerve, peripheral vasoconstriction via the sympathetic NS), but most elect not to explore central integration.

**FIGURE 3:**
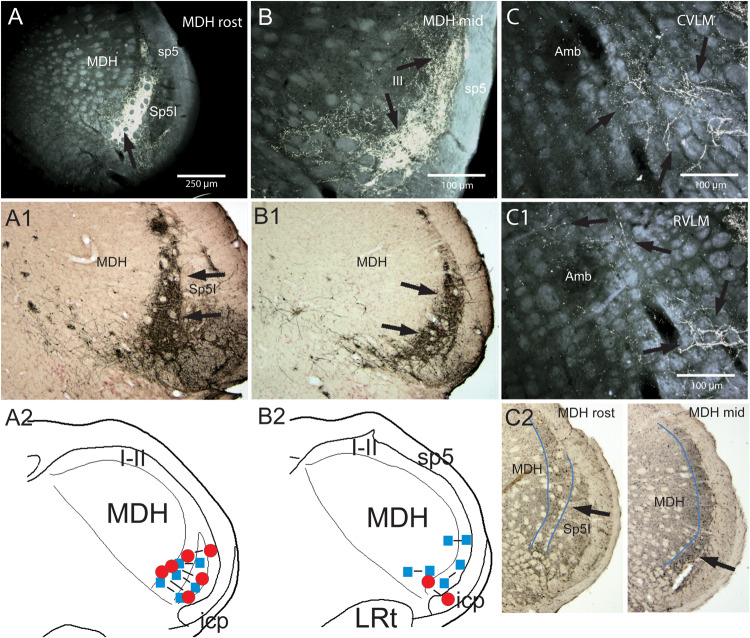
Support for the role of the anterior ethmoidal nerve (AEN) and the medullary dorsal horn of the trigeminal sensory complex as relays for the diving circuit. Figures showing some medullary projections of the AEN, locales where injections into the MDH disrupted the cardiorespiratory responses after nasal stimulation, and distribution of cFos-labeled neurons in the MDH after involuntary submersion. Dense projections are shown in darkfield photomicrographs at two levels of the rostral MDH in its substantia gelatinosa (laminae I and II) after transganglionic transport of an HRP cocktail after its application to the AEN in a rat (**A,B**; arrows; labeled axons/terminals appear bright white), or HSV-1 virus in the muskrat (**A1,B1**; arrows; immunoprecipitate appears dark). Note the similarity of data in the two species using different techniques. The cardiorespiratory responses to stimulating the nasal mucosa (see Figure 1) were blocked by small bilateral injections of either lidocaine (blue squares) or kyurenate (red circles) made into similar areas of the muskrat **(A2,B2)**. The role of the MDH as a relay for the diving response was also supported by cFos in similar areas of the rat after underwater submersion (**C2**, arrows). Extratrigeminal projections of the AEN also were noted in the lateral reticular formation at levels of the CVLM (**C**, arrows) and RVLM (**C1**, arrows). Both areas are important for modulating cardiovascular activity and these projections suggest direct somato-autonomic connectivity. Location of the bilateral injections of blocking solutions is coupled in **(A,B)**. Abbreviations: Amb, ambiguus nucleus; CVLM, depressor area of caudal medulla; LRt, lateral reticular nucleus; MDH, medullary dorsal horn; Sp5I, nucleus of the spinal tract of the trigeminal nerve, interpolar part; RVLM, pressor area of rostral medulla; icp, inferior cerebellar peduncle; sp5, spinal tract of the trigeminal nerve. Figure is compiled from others in Neuroscience, 141, Panneton et al., Brainstem projections from recipient zones of the anterior ethmoidal nerve in the medullary dorsal horn, 889–906 (2006); Physiology 28, Panneton, The mammalian diving response: an enigmatic reflex to preserve life?, 284–297 (2013), and Br. Res. 874, Panneton et al., Trigemino-autonomic connections in the muskrat: the neural substrate for the diving response, 48–65 (2000), with permission. See text for details.

The trigeminal sensory complex is the principal relay for somatosensory afferent fibers innervating structures in the head (Beckstead and Norgren, 1979; Marfurt, 1981; Panneton and Burton, 1981; Matesz, 1983; Shigenaga et al., 1986; Marfurt and Rajchert, 1991; Panneton et al., 2017). The central projections of the AEN have been studied with transganglionic transport techniques in the cat (Lucier and Egizii, 1986), muskrat (Panneton, 1991a), guinea pig (Segade, 2003), and rat (Panneton et al., 2006; Hollandsworth et al., 2009), or after mucosal injections in the rat (Anton and Peppel, 1991; McCulloch et al., 2018). Most of these studies (Lucier and Egizii, 1986; Anton and Peppel, 1991; Panneton, 1991a; Panneton et al., 2006; McCulloch et al., 2018) show dense reaction product in superficial laminae of the subnucleus caudalis of the spinal trigeminal nucleus (currently called the MDH) (Figures 3A,B), while projections to more rostral parts of the trigeminal sensory complex also were shown in some reports (Panneton, 1991a; Panneton et al., 2006). Panneton et al. (2000) further showed transganglionic transport of herpes simplex virus (HSV-1, strain 29) from the AEN to similar areas of the trigeminal sensory complex (Figures 3A1,B1) as well as transneuronal projections to brainstem autonomic nuclei in the muskrat (Figures 5A4,B4,C4). The central projections of the infraorbital nerve partially overlap those of the AEN in the rostral MDH (Panneton et al., 2010c) and must be important since the DR in awake behaving rats is maintained despite cutting the AEN bilaterally (Chotiyanonta et al., 2013; McCulloch et al., 2016; McCulloch and DiNovo, 2018).

The MDH is an important relay in autonomic reflexes such as the DR (Panneton, 1991b; Panneton and Yavari, 1995; Yavari et al., 1996), trigeminal depressor response (Kumada et al., 1975, 1977; Terui et al., 1981; Yu and Blessing, 1998), oculocardiac reflex (Gandevia et al., 1978), and adrenal cortical function (Bereiter and Gann, 1988a, b, 1989; Bereiter et al., 1990; Bereiter and Benetti, 1991; Lu and Bereiter, 1991; Bereiter, 1993). Indeed, underwater submersion activates numerous neurons immunolabeled with cFos in the MDH (McCulloch, 2005; Panneton et al., 2010a, 2012a; McCulloch et al., 2016) in locations similar to the termination of primary afferent fibers contained within the AEN (Figure 3C2). Moreover, Panneton (1991b) and Panneton and Yavari (1995) showed that small injections into similar areas (Figures 3A2,B2) of either lidocaine or kynurenate, both of which block synaptic transmission, selectively inhibited the cardiorespiratory sequelae to nasal stimulation. It should be noted, however, that the AEN also has extratrigeminal reticular projections (Figures 3C,C1) which are probably important for the cardiovascular responses in diving.

## Respiration

**FIGURE 4:**
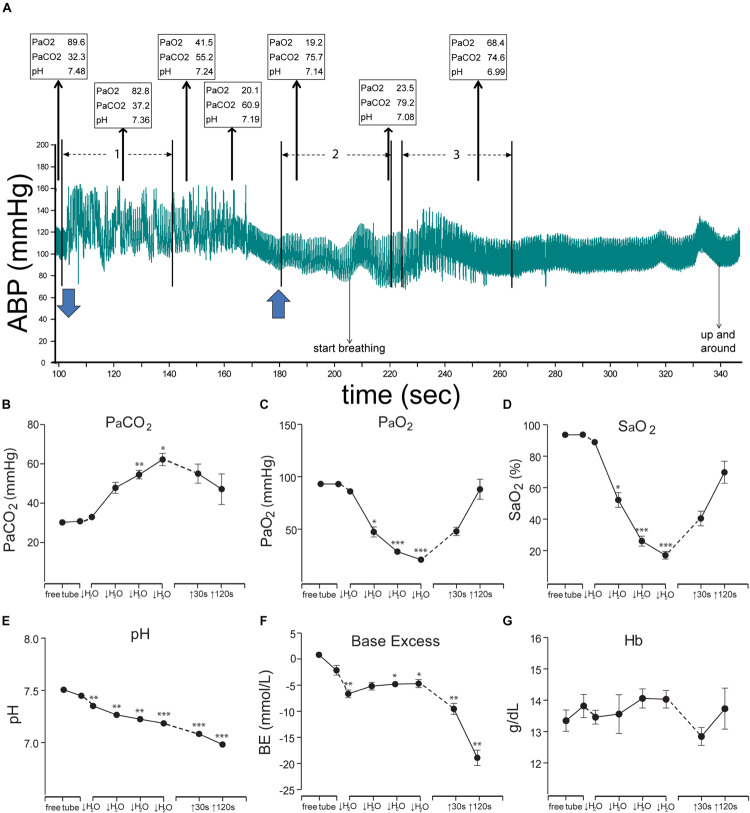
Figures illustrating the cardiovascular responses and the resultant blood chemistry to forced submersions of rats. A bradycardia and increase in ABP was seen approximately for the first minute of submersion **(A)**; numerous ectopic beats are evident with increased pulse pressure (see expanded view in Figure 11). Blood chemistry changed radically during the period of submersion **(B–G)**, but the rats remained apneic despite such changes. Thin arrows oriented upward in **A** show (in boxes) the P_a_O_2_, P_a_CO_2_, and pH of blood withdrawn over time from the submerged rat, while blue arrows at the bottom indicate time underwater. Note the extreme hypercapnia, hypoxia, and acidosis during the apnea. All the chemical indicators in the blood however suggested the rats should breathe vigorously, but the rats did not, nor did they drown. We speculate the apnea is refractory to gross changes in blood gases and is prolonged during diving, perhaps due to the activation of putative chemoreceptors on the ventral medullary surface (see Figures 6, 7C). These studies prove the homeostatic respiratory chemoreceptor reflex is inhibited during underwater submersion. ^∗^*P* < 0.05, ^∗∗^*P* < 0.01, ^∗∗∗^*P* < 0.001. Figures are reprinted from J. Appl. Physiol., 109, Panneton et al., Cardiorespiratory and neural consequences of rats brought past their aerobic dive limit, 1256–1269 (2010), with permission. See Panneton et al. (2010a), for more details.

All mammals submerged underwater, either voluntarily or involuntarily, became apneic and remain apneic, despite submersions exceeding their aerobic dive limit (Panneton et al., 2010a). However, such prolonged apneas (or breath-holds) are not maintained with long nasal stimulations in anesthetized muskrats. Nevertheless, most marine mammals work within their aerobic dive limit (Kooyman, 1985; Burns and Castellini, 1996; Ponganis et al., 1997), a metabolic threshold where diving duration goes beyond intrinsic oxygen stores and is marked by blood lactate concentration increasing above resting levels (Kooyman et al., 1980). An important question is how the prolonged apneas are maintained despite gross alterations in blood chemistry (Figures 4B–G) that normally *increase* ventilation. The neuronal circuitry driving respiration is complexly organized and its efficiency in fulfilling physiological needs is not fully understood (Feldman et al., 2013). Nevertheless, a reflex apnea is induced with either underwater submersion or nasal stimulation despite truncating the brain at the pontomedullary junction (Figure 2B). While it seems reasonable to believe influences over reflex behavior are manifested by many suprabulbar neurons, including those important in apneic reflexes and breath-holds (Dutschmann and Herbert, 1996, 1997, 1998b, 1999; Chamberlin and Saper, 1998; Radulovacki et al., 2003, 2004; Topchiy et al., 2009), it is likely that they are modulatory rather an intrinsic part of the diving reflex circuit. This is especially important to consider when studying the DR in species high in neural hierarchies, e.g., marine mammals and humans, who have considerable volitional control over respiration.

Indeed, rhythmic depolarizations similar to respiration persists in many slice or brainstem-spinal preparations of only the medulla (Bianchi et al., 1995; Rekling and Feldman, 1998; Feldman et al., 2013), and much information has been garnered from such preparations. The ventral respiratory column (Feldman, 1986; Benarroch, 2007) holds many respiratory neurons and one part of it, the pre-Bötzinger complex, is where many neurons generating respiratory rhythm lie (Bianchi et al., 1995; Rekling and Feldman, 1998; Feldman et al., 2013). Dutschmann and Paton (2002) showed that inspiratory neurons ceased firing and were hyperpolarized while post-inspiratory neurons depolarized and discharged persistently after electrical stimulation of the AEN in a working heart-brainstem preparation. This novel preparation may be advantageous for future investigations of the apnea related to diving, since the “preparation” is unanesthetized but has intact and functioning cardiac and respiratory systems. Reflecting on the caveats associated with unnatural electrical stimulation of nerves, however, perhaps it would be worthwhile to determine if these preparations could also induce a DR with an immersion of the snout in water.

**FIGURE 5:**
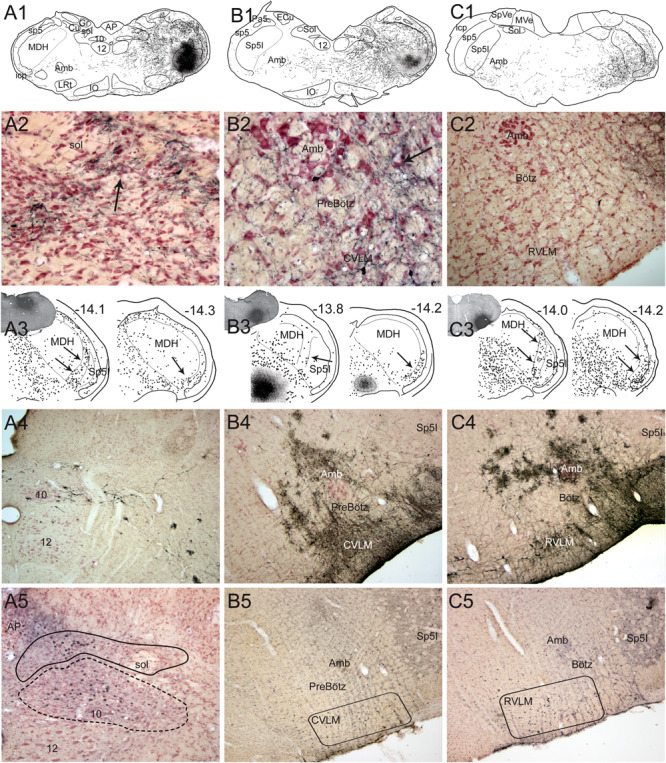
Neuroanatomical data implicating brainstem loci important for the diving response. Figures from rats (**1–3** in **A–C**) and muskrats (**4** in **A–C**) illustrating potential brainstem circuits driving the diving response. Injections of BDA were placed in the MDH **(A1)** where primary afferent fibers in the AEN project, where transganglionic transport of HSV-1 virus was found, where the DR could be reversibly inhibited, and where cFos labeled neurons were found (see Figure 3). Labeled fibers packed into the ventrolateral subnucleus of the nucleus tractus solitarius (Sol; **A2**, arrow) and extended toward the lateral part of the dorsal motor nucleus of the vagus. This projection was confirmed after injection of fluorogold into the Sol (**A3**, insert); note numerous retrogradely labeled neurons (dots) in the substantia gelatinosa of the MDH rostrally (-14.1; arrows) and more caudally (-14.3; arrow) mimicking the location of data seen in Figure 3. Terminal-like label was also seen in sections through the preBötzinger (PreBötz) area and CVLM **(B1,B2)** after MDH injections of BDA **(A1)** and confirmed by retrograde cases **(B3)**. Sections through the more rostral Bötzinger (Bötz) complex and RVLM had very few large fibers labeled with BDA but numerous small fibers **(C2)**. The origin of the projections was confirmed with retrograde analysis (**C3**, arrows) showing numerous neurons in the part of the MDH known to be important for diving behavior. cFos studies, considered functional neuroanatomy, suggest the reticular areas also are activated during underwater submergence (**A5–C5**; darkened nuclei represent activated neurons). Other neuroanatomical data from transneuronal transport of HSV-1 virus **(A4–C4)** injected into the AEN, suggest these areas are linked to the AEN as well as underwater submersion (see Panneton et al., 2000 for details). However, mismatches of label between functional and tract-tracing approaches in the subnuclei of Sol suggest the fibers seen with tract-tracing techniques have functions other than diving behavior. Also, there were few neurons labeled with Fos in the ventral respiratory column, perhaps since respiration is inhibited (the apnea) and Fos only labels activated cells (see text for discussion). Abbreviations: AP, area postrema; Cu, cuneate nucleus; Gr, gracile nucleus; IO, inferior olive nucleus; MVe, medial vestibular nucleus; Sol, nucleus tractus solitarii; SpVe, spinal vestibular nucleus; py, pyramidal tract; sol, solitary tract; 10, dorsal nucleus of the vagus nerve; 12, hypoglossal nucleus; 12n, hypoglossal nerve. See Figure 3 for other abbreviations. Figure is compiled from others in Neuroscience, 141, Panneton et al., Brainstem projections from recipient zones of the anterior ethmoidal nerve in the medullary dorsal horn, 889–906 (2006); and Br. Res. 874, Panneton et al., Trigemino-autonomic connections in the muskrat: the neural substrate for the diving response, 48–65 (2000), with permission.

Neuroanatomical projections from the MDH (Panneton et al., 2000, 2006) are relatively dense to caudal parts of the ventral respiratory column where expiratory neurons dominate (Figure 5A1). Projections from the MDH also are seen near the pre-Bötzinger complex (Figures 5B1–B4). However, there are very few neurons labeled with cFos in the ventral respiratory column (Figures 5B5,C5, 6), considered the area just ventral to the nucleus ambiguus, after underwater submersion (Panneton et al., 2010a, 2012a). This is perhaps due to the apnea induced with underwater submergence—inhibited neurons rarely, if ever, show Fos label because their inhibition precludes activation.

**FIGURE 6:**
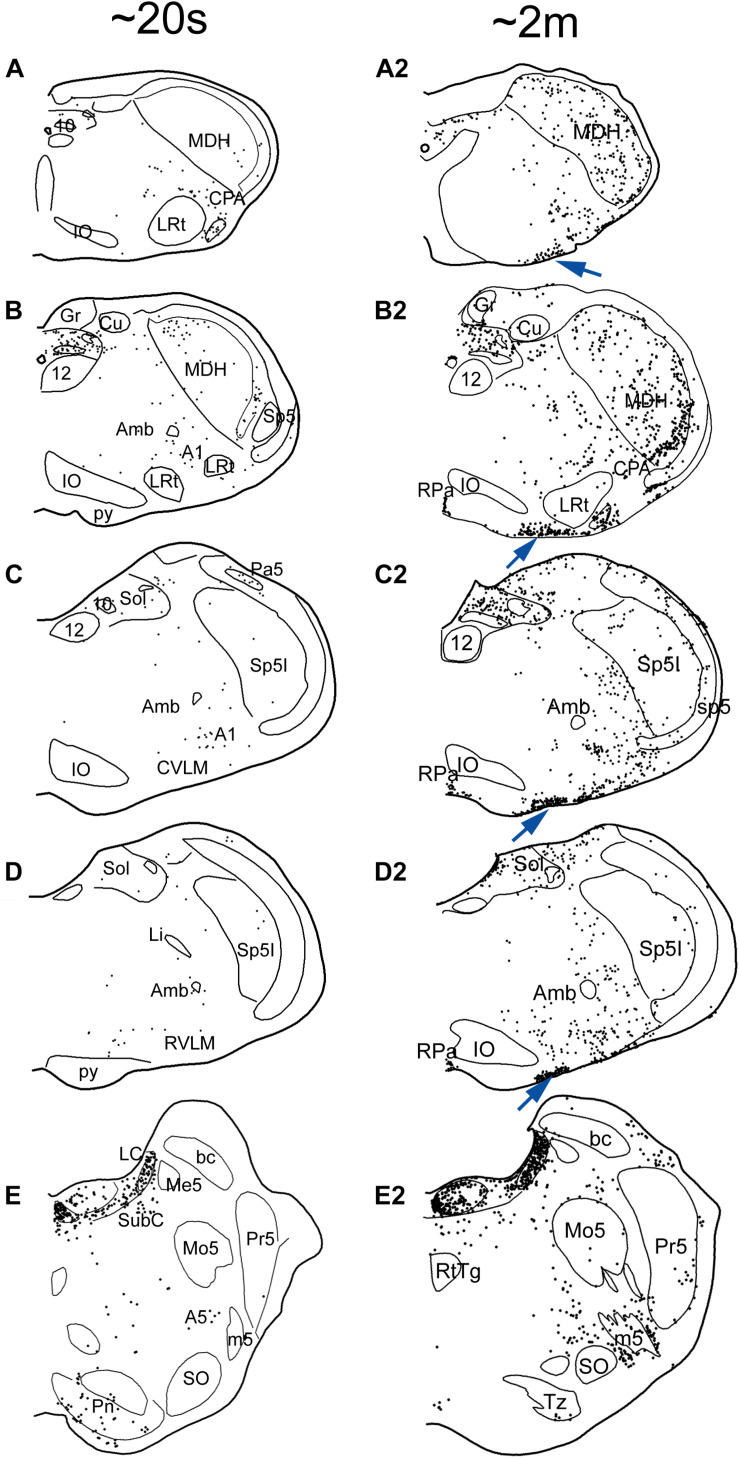
Line drawings comparing the distribution of cFos immunolabeling in the brainstem of rats after a single trial of voluntary submersion (**A–E**; see Panneton et al., 2012a) to that of a single prolonged submergence of a rat brought beyond its aerobic dive limit (**A2–E2**; see Panneton et al., 2010a). The density of immunolabeled cells in the MDH, CVLM, RVLM, and NTS after a single brief immersion (left column) is greatly increased after the more prolonged submersion (right column). Note, however, the emergence of myriad immunolabeled cells along the ventral surface of the medulla (arrows), most in the epi-pia, only after the prolonged submergence. These putative chemoreceptors could potentiate and prolong the apnea of diving via interaction with somatostatin neurons (Figure 7). See previous figures for abbreviations. Figures are reprinted from J. Appl. Physiol., 109, Panneton et al., Cardiorespiratory and neural consequences of rats brought past their aerobic dive limit, 1256–1269 (2010), with permission.

The pre-Bötzinger complex contains several types of neurons, including those marked by somatostatin (Stornetta et al., 2003; Tan et al., 2008; Cui et al., 2016), a peptide, which acts as an inhibitory respiratory modulator (Ramirez-Jarquin et al., 2012). Acute silencing of such somatostatin neurons results in persistent apnea (Tan et al., 2008) in awake mice. Moreover, somatostatin infusion in humans greatly reduced the acute hypoxia ventilatory response, as well as the acute hypercapnic ventilatory response (Pandit et al., 2014), thus blunting the respiratory chemoreceptor response. Indeed, somatostatin’s inhibitory effect on respiration was potentiated *in vitro* when the pH of brainstem’s bath was lowered from 7.4 to 7.3 (Llona et al., 2004). The pH of blood dropped from ∼7.5 to ∼7.2 during involuntary submergence of rats (see Figure 4E) and continued to drop after they emerged from the water. Thus, somatostatin neurons in the pre-Bötzinger complex may be important for the apnea induced in the DR.

**FIGURE 7:**
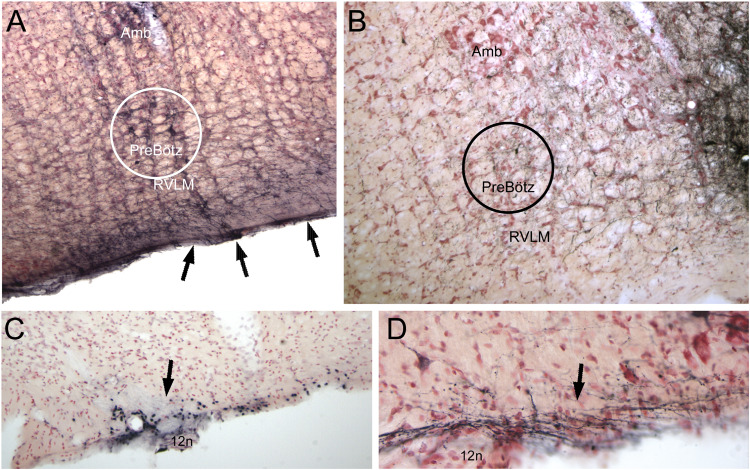
Neuroanatomical data implicating somatostatin neurons in the preBötzinger complex as well as neurons/cells near the ventral medullary surface as important for the diving circuit. Somatostatin neurons in the preBötzinger area have been shown important for apnea (see text for details). In **A**, several somatostatin immunostained neurons of medium size are encircled. Note that putative dendrites from these somatostatin neurons stream to the ventral medullary surface and appear to intertwine among the epi-glial cells found here (arrows). Anterograde transport of BDA after an injection in the ventral MDH is seen over similar medium-sized neurons in **B** (see also Figure 4B2). Neurons/cells near the ventral medullary surface always were immunolabeled with cFos after long submersions (see Figure 6, right column). Those found caudally near the pyramidal decussation (**C**, arrow), often surrounded the exit of the hypoglossal nerve (12n). The far majority of injections of BDA into the ventral MDH also showed small labeled fibers, with boutons, over similar areas (**D**, arrow). The epi-pia on the ventral medulla are linked by gap junctions; we hypothesize a depolarization of similar caudal epi-pia during diving would then rapidly flow rostrally, impinging on the distal dendrites of the apnea-inducing somatostatin neurons in the preBötzinger complex. See text for discussion.

Somatostatin neurons in the pre-Bötzinger complex (Figure 7A) have numerous processes which extend ventrally into the epi-pia on the ventral surface of the medulla. Our data provide two potential routes where such neurons in the preBötzinger complex may be modulated during the DR. The first is via direct projections from neurons in the ventral MDH that receives nasal afferent fibers (Figure 3) to the area of medium-sized neurons where somatostatin neurons lie (Figures 5B2,B4, 7B). [It is of interest that a cluster of neuron in a similar place were activated and immunolabeled for cFos after a prolonged submersion (Figure 6C2), but we unfortunately did not double label these neurons for somatostatin.] A second potential route is via projections from similar injections to the ventral surface of the caudal medulla (Figure 7D), where numerous Fos labeled cells are documented (Figure 7C) after involuntary submersion. These putative respiratory chemoreceptors are linked by gap junctions (Solomon et al., 2001; Dean et al., 2002) and may provide a fast link to somatostatin neurons of the brainstem respiratory network and modulate the apnea induced by underwater submergence. However, the function of neither of these projections is known, highlighting the technical limitations of neuroanatomical techniques. Fos immunohistochemistry fails to label inhibited neurons, while tract-tracing studies offer no insight into functional status. More precise experiments are needed, perhaps with genetically altered mice or working heart-brainstem preparations, to determine the genesis of the apnea in the mammalian DR.

## Heart Rate

**FIGURE 8:**
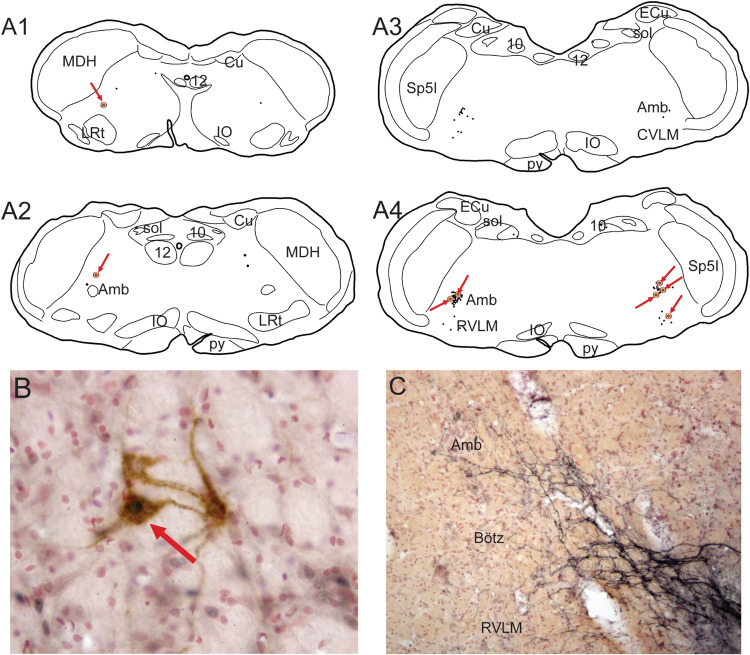
Illustrations showing functional neuroanatomical data from rats that voluntarily dove underwater after pericardial injections of cholera toxin into their pericardial cavities (Panneton et al., 2014) and its retrograde transport to the medulla. All rats showed marked bradycardia and an increase in arterial blood pressure typical of underwater submersion (see Figure 1B). Preganglionic cardiac motoneurons were found caudally mostly in the medullary reticular formation sandwiched between sensory and motor areas (**A1,A2**; arrows), but a few also were noted in the dorsal motor nucleus of the vagus nerve. Single black dots represent such preganglionic cardiac motoneurons labeled solely by cholera toxin. Double-labeled neurons represent preganglionic cardiac motoneurons activated by diving. Double-labeled neurons, marked by black dots labeled by Fos immunohistochemistry encircled by brown cytoplasmic labeling of cholera toxin (**B**, red arrow), were found throughout the ventrolateral medulla, but mostly rostrally (**A1–4**, encircled dots highlighted by red arrows). It is of interest that primary afferent fibers contained within the AEN project *directly* into similar reticular areas (**C**; compare to A4). See legends in Figures 3, 4 for abbreviations and Panneton et al. (2014) for details. Figures are reprinted from Front. Physiol., 5, Panneton et al., Parasympathetic preganglionic cardiac motoneurons labeled after voluntary diving, 8, (2014), with permission.

The dramatic bradycardia seen with underwater submersion, or after stimulation of the AEN or nasal mucosa, is mediated via the vagus nerve (see prodigious review by Ponganis et al., 2017 for their hypothesis). Most preganglionic parasympathetic cardiac motoneurons are found in the external formation of the nucleus ambiguus (Panneton et al., 1996, 2014; Taylor et al., 2001), an area of reticular formation separating sensory and somatic motor nuclei where many preganglionic autonomic neurons occur (Figures 8A1,A2; arrows). More cardiac motoneurons were found more rostrally in the CVLM (Figure 8A3) but most double labeled neurons were found in the RVLM. Double-labeling cardiac motoneurons (Panneton et al., 2014) with cFos after voluntary diving and cholera toxin after retrograde transport from cardiac injections (Figure 8B, arrow) showed that double-labeled neurons were mostly in the rostral medulla (Figure 8A4; red arrows), especially surrounding the compact formation of nucleus ambiguus. Neurons, possibly preganglionic parasympathetic cardiac motoneurons, in similar areas (see Figures 3, 5) are labeled transneuronally after HSV-1 virus injections into the AEN (Panneton et al., 2000), after injections of BDA into the MDH (Panneton et al., 2000, 2006), as well as after transganglionic transport of label in primary afferent fibers of the AEN (Panneton, 1991a; Panneton et al., 2006) (Figure 8C).

Work in *in vitro* brainstem slices show preganglionic parasympathetic cardiac motoneurons are modulated by both glutamatergic (Willis et al., 1996; Mendelowitz, 1998; Neff et al., 1998; Corbett et al., 2003) and GABAergic/glycinergic (Wang et al., 2001, 2003) inputs from the NTS. These cardiac neurons are also modulated by nicotinic cholinoceptors (Wang et al., 2001), which facilitate glutamatergic input to them (Huang et al., 2004), numerous peptides (Agarwal and Calaresu, 1991; Ruggeri et al., 2000; Irnaten et al., 2003; Blinder et al., 2005, 2007), and monoamines (Izzo et al., 1993; Wang and Ramage, 2001; Skinner et al., 2002; Gorini et al., 2009). Indeed, cardiac motoneurons activated by the stimulation of the trigeminal tract are modulated by serotonin (Gorini et al., 2009) and acetylcholine (Gorini et al., 2010) receptors. The bradycardia to nasal stimulation is enhanced when electrical stimulation of the AEN is paired with chemical stimulation of peripheral chemoreceptors (Rozloznik et al., 2009), and was even more potentiated by injections of a 5HT receptor agonist injected into the NTS, implying an integrative function of the NTS in the multimodal mediation of the DR. Such mechanisms may be important in the more prominent bradycardias seen in aquatic animals during deep dives.

Moreover, electrophysiological investigations on postganglionic cardiac motoneurons driven by diving have commenced (McAllen et al., 2011). Cardiac nerves from both the parasympathetic vagus nerve and the sympathetic system are activated during nasal stimulation with formaldehyde vapors in the rabbit (Nalivaiko et al., 2003), and the sympathetic contribution may maintain or enhance cardiac output during the bradycardia (Paton et al., 2005). We speculate that the bradycardia induced by underwater submersion activates cardiac motoneurons directly either by primary afferent fibers from the AEN projecting into the nearby reticular formation (Figures 3C,C1) and/or indirectly via projection neurons from the MDH.

## Arterial Blood Pressure

**FIGURE 9:**
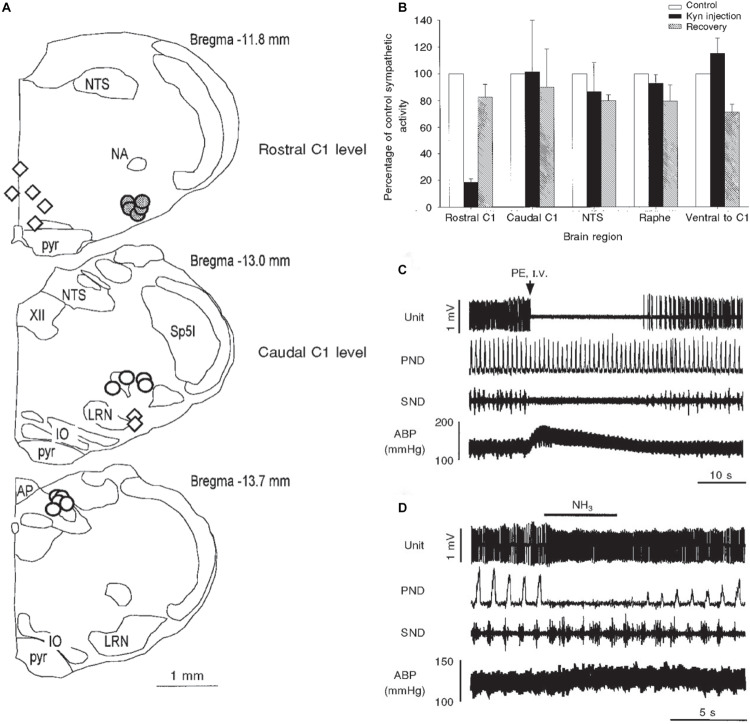
Illustrations mapping physiological data implicating several medullary areas important for the diving response. Bilateral injections of kyurenate made at levels of the rostral C1 area (RVLM), caudal C1 area (CVLM), nucleus tractus solitarii (NTS), and raphe obscuris are plotted in **A**. The effect of nasal stimulation on sympathetic nerve discharge (SND) was unchanged after injections into the CVLM, NTS (open circles) or raphe and ventral to CVLM (open diamonds), but was reduced by 80% after injections into the RVLM, suggesting the RVLM mediates the sympathetic response. Normalized sympathetic responses to nasal stimulation from multiple trials are shown **(B)**; again, only injections into the rostral C1 induced a significant decrease in SND. The electrophysiological responses of a typical single baroreceptive neuron in the RVLM is seen in **C**; note that this unit is *silenced* by increases in blood pressure after phenylephrine administration and SND ceases, but phrenic nerve discharge (PND) is maintained. The same neuron is *excited*, however, after nasal stimulation **(D)**, even with the increase in ABP; the PND is also silenced with nasal stimulation and the SND is increased. 24/39 of similar baroreceptive neurons, normally silent with increases of ABP, actually increased their firing rate by nearly 66% and increased SND by 102%, despite an increase of ABP of 28 ± 2 mmHg. These data suggest the homeostatic baroreceptor reflex is inhibited during diving. Figures are reprinted from J. Physiol., 516, McCulloch et al., The rostral ventrolateral medulla mediates the sympathoactivation produced by chemical stimulation of the nasal mucosa, 471–484 (1999), with permission. See McCulloch et al. (1999a) for more details.

Numerous studies have shown that neurons in the rostral ventrolateral medulla (RVLM) regulate ABP by maintaining sympathetic tone. Moreover, numerous studies have also implicated the RVLM as the brainstem relay to the spinal cord for the baroreceptor reflex (Guyenet, 1990; Schreihofer and Guyenet, 1997; McCulloch et al., 1999b) as well as somatosympathetic reflexes (Stornetta et al., 1989; Burke et al., 2011). The reflex circuitry driving the baroreceptor reflex has been described extensively and involves neurons in the nucleus tractus solitarii (NTS), the caudal ventrolateral medulla (CVLM), and the RVLM (Aicher et al., 2000). The increase in ABP from the induced vasoconstriction after underwater submersion activates the baroreflex, but the baroreceptive circuitry does not overlap that of the diving circuit until the RVLM (McCulloch et al., 1999b). Thus, the neuroanatomical projections from the MDH to the NTS (Figures 5A1–A3), as well as those from the transneuronal transfer of virus from the AEN (Figure 5A4), do not overlap with neurons labeled with cFos after underwater submersion (Figure 5A5). These neuroanatomical tract-tracing techniques label fibers/neurons indiscriminate of function; we believe MDH projections to the NTS are labeling fibers/neurons more associated with pain pathways versus those in the diving circuit. Moreover, bilateral injections of the excitatory amino acid receptor antagonist kynurenate made into the dorsolateral subnucleus of the NTS or the CVLM, where the baroreceptive neurons lie, greatly attenuated the baroreflex but failed to modify responses from nasal stimulation (McCulloch et al., 1999b) (Figure 9A). This view is contrary to that of others (Huang et al., 1991; Dutschmann and Herbert, 1998b) who concluded the NTS modulates diving behavior. However, injections into the RVLM greatly reduced effects of nasal stimulation on sympathetic nerve discharge but not that from baroreflex activation (Figures 9A,B). The lack of change in baroreceptor modulation of sympathetic activity after the RVLM injections of kainate is explained by the predominate GABAergic input from the CVLM to the RVLM, while blocking of RVLM activation after nasal stimulation suggests excitatory amino acids synaptically drive this projection.

**FIGURE 10:**
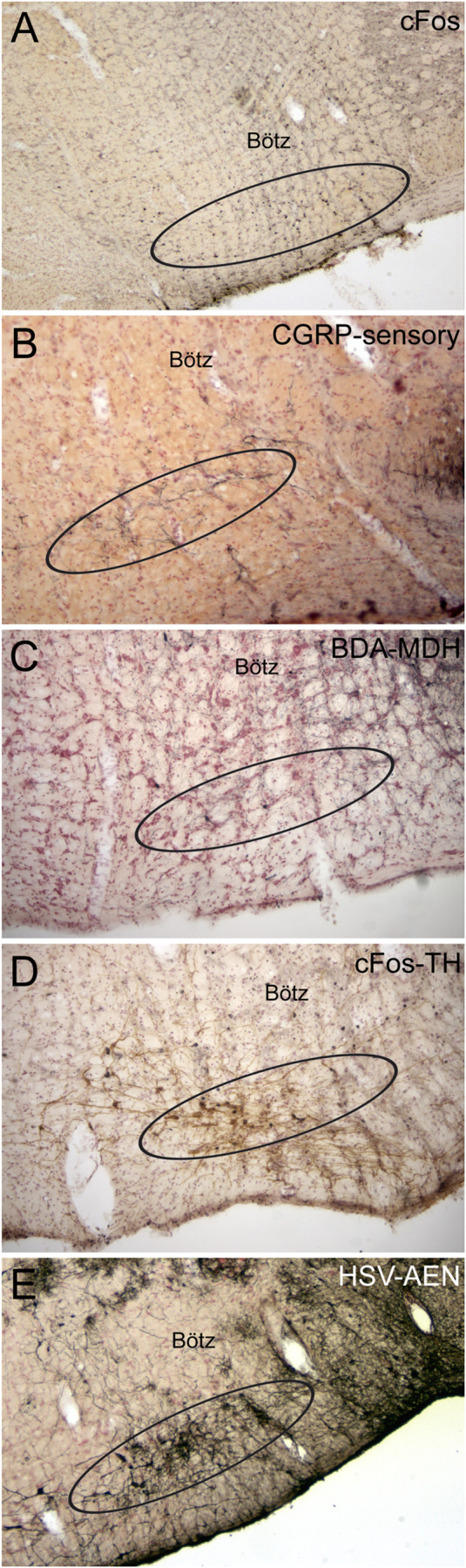
Photomicrographs of neuroanatomical data implicating the RVLM as an important link in the circuit driving the diving response. Ovals drawn ventral to the Bötzinger complex (Bötz) demarcate our definition of the RVLM in **A–E**. Numerous neurons always were immunolabeled with cFos in the RVLM after underwater submersion **(A)**, and many of these neurons were also double labeled for tyrosine hydroxylase **(D)**, suggesting that both noradrenergic as well as non-noradrenergic neurons are activated. We have shown (see Figure 8C) that the anterior ethmoidal nerve projects *directly* to the RVLM; **B** shows that many of these fibers are also immunoreactive to CGRP (see Panneton and Gan, 2014 for discussion). The RVLM also receives *indirect* projections from nasal areas of the MDH. Two tracing techniques, the anterograde transport of BDA after an injection in the ventral MDH of the rat (**C**; see Panneton et al., 2006 for details) and the transneuronal transport of HSV-1 virus in a muskrat (**E**; see Panneton et al., 2000 for details) suggest this is the case.

The RVLM contains the rostral C1 adrenergic cell group (Ruggiero et al., 1985) that provides bulbospinal projections to the intermediolateral cell column in the spinal cord; many such neurons are activated by underwater submersion (Figures 5C5, 10D) (McCulloch and Panneton, 2003). However, both adrenergic and non-adrenergic spinally projecting neurons in the RVLM are responsive to nasal stimulation (McCulloch et al., 1999b). Moreover, 62% of the same baroreceptive RVLM neurons normally silenced by increases in ABP are *excited* by nasal stimulation despite increases in ABP (Figures 9C,D; McCulloch et al., 1999b), suggesting that the homeostatic baroreceptor reflex is overridden. Several lines of neuroanatomical evidence also suggest that these RVLM neurons are important in the DR, including their activation of cFos (Figure 10A) after diving, the overlap of CGRP fibers (from primary afferent fibers) (Figure 10B), and the overlap of projections from the MDH (Figures 10C,E). These bulbospinal neurons could get input from somatosensory neurons either directly from primary afferent fibers of the AEN into the ventrolateral reticular formation (Figures 3C,C1) or relay from non-baroreceptive neurons in the CVLM or from the MDH.

## Suprabulbar Control of the Diving Response

We were initially impressed by reading many years ago that a seal showed an abrupt and dramatic bradycardia *prior* to underwater submergence and a tachycardia *prior* to emersion (Casson and Ronald, 1975). Also, differences in heart rate of marine mammals diving voluntarily show that the DR is more variable and less intense than during involuntary dives (Kooyman and Campbell, 1972; Hill et al., 1987; Jobsis et al., 2001). Similarly, the hemodynamic responses to “forced” submersions when mammals are involuntarily “dunked” underwater (Koppányi et al., 1929; Scholander, 1963; Tchobroutsky et al., 1969; Dykes, 1974; Lin, 1974; Lin and Baker, 1975; Martner et al., 1977; Drummond and Jones, 1979; Jones et al., 1982; Hill et al., 1987; McCulloch and Jones, 1990; Jobsis et al., 2001; Panneton et al., 2010b) are subtly dissimilar to the hemodynamics of voluntary diving (Drummond and Jones, 1979; Kooyman, 1989; McCulloch and Jones, 1990; Rybka and McCulloch, 2006; Panneton et al., 2010b). This suggests that marine mammals may have “control” over their “autonomic” NSs, which is considered taboo by many teachers of physiology but certainly has adherents (Blix et al., 2010). The compendium by Houser (2018), as well as that of Elmegaard et al. (2016) cite copious examples documenting volitional control of heart rate by marine mammals, supporting this case. However, there are numerous factors controlling the HR in diving mammals including temperature, apnea, and submergence duration and depth, as well as exercise intensity (Davis and Williams, 2012; Noren et al., 2012; McDonald and Ponganis, 2014; Williams et al., 2015; Kaczmarek et al., 2018; McDonald et al., 2018). For example, the HR increases with exercise (a sympathetic response) working against an increasing bradycardia (a parasympathetic response) with depth. Moreover, recent data on seals using non-invasive infrared spectroscopy show these animals routinely exhibit preparatory peripheral vasoconstriction accompanied by increased cerebral blood volume approximately 15 s *before* submersion (McKnight et al., 2019). These anticipatory adjustments confirm that blood redistribution in seals also is under some degree of cognitive control that precedes the mammalian dive response. Thus, while respiration is under volitional control in higher mammals, these data also suggest that higher marine mammals can also control their cardiovascular systems volitionally.

Thus, it is possible that preventing an organism from deciding its own fate by involuntary submersion may induce both fear and stress, and these emotions may alter normal reflex responses. Studies on terrestrial animals have shown both significant bradycardia and increases in ABP during extreme fear (Gabrielsen et al., 1977; Smith and Woodruff, 1980; Smith et al., 1981; Smith and Tobey, 1983; Carrive, 2000; Zhang et al., 2004), similar to that seen during diving. However, the bradycardia in all of our studies on rats has been locked tightly to the time submerged, and immediately returned to normal after exiting the water (Figures 1B,C). It is noteworthy, however, that changes in the HR and the ABP were more varied in dunked naïve rats (Figure 1C2) and there were more arrhythmias (Figures 4A1, 11; de Burgh Daly, 1984). We and others noted similar changes previously (Byku et al., 2004; McCulloch et al., 2010) and McCulloch et al. (2010) concluded that forced submergence is stressful to both naïve and trained rats but voluntary diving in trained rats is no more stressful than being handled by humans. While it is generally accepted that the bradycardia of voluntary diving is vagally mediated and dominant, forced underwater submersion stresses the animal and may also activate suprabulbar neurons influencing the sympathetic NS. Many have noted that coactivation of both parasympathetic and sympathetic cardiac nerves induces cardiac arrhythmias (Paton et al., 2005; Shattock and Tipton, 2012). The arrhythmias during forced diving possibly induced “fear” or stress, activating the sympathetic NS and countering the bradycardia of underwater submersion.

**FIGURE 11:**
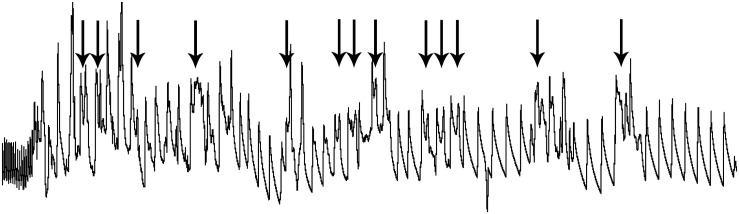
An expanded trace of ABP in a rat involuntarily submerged underwater. The involuntary submersion induces stress in the naïve animal, inducing sympathetic discharge. The marked slowing of the pulse on the left of the trace shows the remarkable bradycardia, the result of parasympathetic discharge, resulting as soon as the rat’s nose is submerged. Arrows above the trace illustrate the confound of ectopic beats, creating arrhythmias, when untrained rats are involuntarily submerged. Such arrhythmias are commonly seen in deep-diving marine mammals and elite human divers and are thought to result from the competitive sympathetic and parasympathetic influences over heart rate. Such “diving” rodents may prove valuable as a tool to study cardiac arrhythmias. Figure is reprinted from J. Appl. Physiol., 109, Panneton et al., Cardiorespiratory and neural consequences of rats brought past their aerobic dive limit, 1256–1269 (2010), with permission.

Seals often show either little bradycardia when diving voluntarily (Kooyman and Campbell, 1972), may reduce heart rate in anticipation of underwater submersion (Casson and Ronald, 1975), induce a bradycardia to non-somatic stimulation (Irving et al., 1942), or an anticipatory tachycardia prior to emerging (Casson and Ronald, 1975; Blix and Folkow, 1983). Sea lions conditioned to adjust their autonomic NSs to auditory or visual commands suggest they may “will” the bradycardia (Ridgeway et al., 1975; Ponganis et al., 1997) from suprabulbar sites. Such premature autonomic behavior in the diving laboratory rat has not been published, however, suggesting that cortical/suprabulbar influences on the DR in the rat is minimal. It is of interest that cetaceans and pinnipeds, considered intelligent species by most, have brains that approach humans’ brains in complexity with highly convoluted cortices (Marino, 1998; Marino et al., 2000, 2001, 2004; Hof et al., 2005; Eriksen and Pakkenberg, 2007; Hof and Van der Gucht, 2007); such complexity overwhelms that of lissencephalic rodent brains. We suggest that the DR has but minimal suprabulbar modulation in rodents, but suprabulbar neurons in higher species, perhaps those in the neocortex, may indeed direct autonomic behaviors seen in the DR. Perhaps the DR is analogous to the blink reflex, a reflex endemic in all mammals. The blink reflex has no suprabulbar control in lower species similar to that in neonatal humans. But as humans’ age and their neocortices mature, they can control their blink reflex volitionally and produce “winking” behavior. Intelligent marine mammals may have harnessed diving behavior similarly.

## The Diving Response in the Human

Diving behavior is well documented in humans (Ferrigno et al., 1997; Ferretti, 2001; Foster and Sheel, 2005; Lindholm and Lundgren, 2009). However, metrics such as the HR are more variable in adults (Olsen et al., 1962; Hiebert and Burch, 2003; Caspers et al., 2011) than in infants (Goksör et al., 2002). The DR, with its elevated activation of vagal cardiac nerves, long has been acknowledged as a treatment for paroxysmal atrial tachycardia (Wildenthal et al., 1975; Gooden, 1982) by normalizing sinus rhythm. Moreover, cases of “cold water drowning” in humans, where children lie submerged underwater for prolonged periods, but recover basically unharmed, have been documented numerous times (Hayward et al., 1984; Golden et al., 1997; Xu et al., 1999; Giesbrecht, 2000). Thus, perhaps in these cases, a DR induces a persistent apnea, saving these victims from inhaling water and drowning.

The powerful DR has also been suggested to be deleterious to the human condition. For example, the DR has been implicated in the etiology of sudden infant death syndrome (SIDS) (Lobban, 1991, 1995; Matturri et al., 2005), where neonates apparently become apneic and die without pathology. Epidemiological data suggest that rebreathing asphyxial gases (mostly carbon dioxide), smoking, and reduced heat loss are important risk factors in SIDS (Kemp, 1996; Leiter and Böhm, 2007; Mitchell, 2009). It is of interest that others have noted the increased prevalence of infections of the upper respiratory tract in SIDS victims (Blackwell and Weir, 1999; Blackwell et al., 1999; Harrison et al., 1999; Molony et al., 1999; Morris, 1999; Rambaud et al., 1999; Goldwater, 2017). Such infections produce inflammatory mediators (Guntheroth, 1989; Lindgren and Grogaard, 1996), which sensitize C-fibers (Reeh et al., 1986; Handwerker et al., 1991; Lee and Widdicombe, 2001) and lower activation thresholds. Indeed, we have shown that small diameter fibers densely innervate the nasal mucosa via the AEN (McCulloch et al., 1999a), while others show inflammatory mediators in the upper respiratory tract promote apnea (Lindgren and Grogaard, 1996). It is of interest that the solitary chemosensory cells found in the anterior nasal mucosa and innervated by small diameter fibers of the trigeminal nerve (Tizzano et al., 2010), probably the AEN, are activated by acyl-homoserine lactones produced by Gram-negative bacteria. Activation of these chemosensory cells also promotes an apnea. Moreover, nasal applications of both smoke (White et al., 1974; Kobayashi et al., 1999; Ho and Kou, 2000) and carbon dioxide (Yavari et al., 1996), both risk factors for SIDS, induce the DR while involuntary submersion induces apneas beyond the aerobic dive threshold (Panneton et al., 2010a), suggesting perhaps the persisting apnea could induce death. Perhaps the DR is induced in infected SIDS victims who nasally rebreathe high levels of CO_2_ (Sakai et al., 2009) so that they hold their breath until they die. A plethora of citations providing background implicating the mammalian DR in sudden cardiac death, arrhythmias, and SIDS in the human clinical literature are found in the theoretical dissertations of Vincenzi (2019) and Vega (2018).

The bradycardia induced in diving humans often is combined with arrhythmias (Scholander et al., 1962; Lindholm and Lundgren, 2009; Shattock and Tipton, 2012), possibly mimicking the yin and yang of cardiac autonomic control seen in rodents (Figure 11). The dual activation of both systems is hypothesized to induce the numerous arrhythmias seen in deep dives in marine mammals. Forced submersion of rodents may provide a model to study these arrhythmias further. The theory of “autonomic conflict” that develops during underwater submersion in cold water, e.g., the activation of both parasympathetic and sympathetic cardiac nerves, may account for the genesis of cardiac arrhythmias and dysrhythmias seen during diving (Shattock and Tipton, 2012; Bierens et al., 2016). The fact that ectopic beats can be generated during diving experiments in rats (Figure 11) might be utilized to test therapies designed to quench these arrhythmias. While autonomic conflict often results in arrhythmias, fatal arrhythmias are much less common and usually coupled with predisposing factors including ischemic heart disease, long QT, channelopathies, and atherosclerosis.

The power of the DR might also be harnessed to combat other human maladies. Cerebral blood flow is significantly increased in humans by inducing a DR (Brown et al., 2003; Kjeld et al., 2009), similar to that of rodents (Irving, 1938; Ollenberger and West, 1998a,b; Ollenberger et al., 1998) and seals (Zapol et al., 1979; Blix and Folkow, 1983; Odden et al., 1999; Tift and Ponganis, 2019), probably in an effort to oxygenate this necessary organ. It is of interest that a cerebral *hypotension* precedes migraine headaches (Olesen, 1991; Thomsen et al., 1995); perhaps inducing a DR in patients experiencing a prefatory aura could alter such cerebrovascular dysregulation and prevent migraines. Using the DR to mitigate certain migraine headaches could be a natural, inexpensive remedy. The DR might also provide therapy in stroke and hemorrhagic shock by increasing cerebral blood flow (Golonov et al., 2016; Chiluwal et al., 2017). Perhaps this feature of the DR could be utilized to reduce the ischemic penumbra and infarct volume due to stroke (Pan et al., 2007; Golonov et al., 2016; Chiluwal et al., 2017). The penumbra describes compromised brain tissue with a decreased oxygen supply which may eventually become necrotic. Increasing blood flow to the penumbra after stroke during a DR may nourish the deprived cells enough to prevent further death. Moreover, the DR has been implicated in sudden unexpected death in epilepsy (Stewart, 2018; Vega, 2018), and experimental studies utilizing nasopharyngeal irrigation concluded that seizure-associated central apnea and the DR share a common neural basis and may reflect an attempt by brainstem networks to protect core physiology during seizure activity (Villiere et al., 2017; Stewart, 2018; Mooney et al., 2019).

Little is known of the neural circuitry driving the DR in humans but behaviors that serve basic vegetative functions are usually less complex and more uniform across species, so we suspect that much known from the reflex circuit of a rodent, or the unexplored circuits of marine mammals, would also apply to humans. Moreover, the fact that the more neurally developed marine mammals can “control” their HR’s at will could perhaps be exploited with techniques designed to show which higher levels of the brain are activated during this autonomic control. Seals and dolphins are increasingly being trained for the study of diving behaviors, including HR regulation. Perhaps they could be trained to perform such feats under a functional MRI or PET scan, an expensive experiment but probably would detail cortical areas directing this control over the autonomic NS. Similar efforts (e.g., fMRI) could be performed on humans, assuming some adult humans can be trained to induce a reliable DR, perhaps with biofeedback techniques, to gain volitional control over their autonomic NS. Indeed, reports on these lines are developing (Abukonna et al., 2013; Jones et al., 2015). Such mind–body interactions could be utilized to control affective symptoms of anxiety in humans (Jones et al., 2015), as well as a method to induce general relaxation for mitigating stress, a malady afflicting an overwhelming number of humans.

As current technology refines and new technologies are born, new discoveries are forthcoming concerning the enigmatic mammalian DR. Indeed, genetic studies are now underway illustrating how diving mammals, including humans, have adapted to their anoxic underwater environments (Fabrizius et al., 2016; Baranova et al., 2017; Hoff et al., 2017; Ilardo et al., 2018; Zhou et al., 2018; Xu et al., 2019). Introduction of the DR in the mouse (Hult et al., 2019) provides opportunity for an entirely novel set of techniques for genetic manipulation of neurons. Utilization of these data on the DR in rodents thus provides practical animal models for study of the mechanisms driving the response, data from which could be applied to humans.

## Summary and Perspectives

**FIGURE 12:**
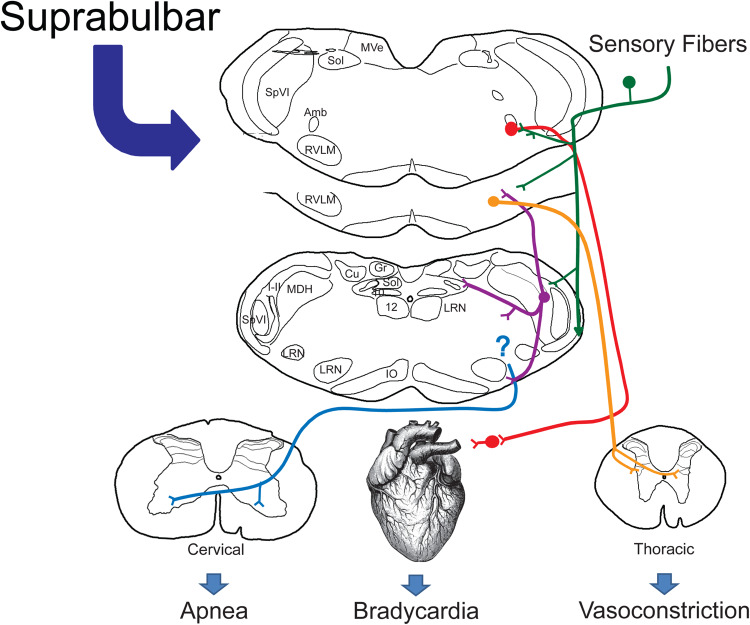
Proposed brainstem circuitry mediating the cardiorespiratory depression with underwater submersion, e.g., the mammalian diving response. We propose direct primary afferent projections from nasal and paranasal areas (green lines) both to the MDH for relay and to the lateral reticular formation, where cardiac motoneurons and sympathetic interneurons lie. We know that the DR can be blocked in the MDH (Figure 3) and neurons from this area project (purple lines) to the CVLM, the RVLM (including sympathetic interneurons and respiratory rhythm-generating neurons), and the ventral medullary surface, where respiratory chemosensitive neurons are found (see text for discussion). Although the location of respiratory neurons sending inhibitory signals to the phrenic motor nucleus in the cervical spinal cord (blue lines) is unknown, the respiratory inhibition may be the result of inhibition of the respiratory pattern generator. The presympathetic neurons in the RVLM project to the intermediolateral cell column of the thoracic spinal cord and are important for mediating the peripheral vasoconstriction (orange lines) during diving. While preganglionic cardiac motoneurons are found throughout the medulla, most neurons double-labeled after diving were near to the compact formation of the nucleus ambiguus juxtaposed to primary afferent fibers, many from the AEN. We propose that these neurons (red lines) project to postganglionic neurons near the heart, inducing the bradycardia seen in the diving response. We suggest this relatively simple but well-organized circuit orchestrates the automatic reflex responses (100% of our rodents 100% of the time show a diving response to underwater submersion). We also propose that such a circuit provides the substrate upon which suprabulbar neurons impinge, allowing higher mammals to willfully control their diving response.

The DR is indeed a dramatic perturbation of normal function, altering basic homeostatic mechanisms to fit physiological needs. This review emphasizes both the reflex nature of the DR and its neuronal circuitry maintained in the medulla and spinal cord, like numerous other reflexes. Many stimuli affecting paranasal areas initiate the DR, and nerves innervating these areas serve as its afferent limb. We suggest sensory fibers of the AEN projecting to the MDH (Figure 12; green lines) mediate much of the DR and noted relays from the MDH to neuronal ensembles driving respiration, heart rate, and vasoconstriction (Figure 12; purple lines). Although only sparse projections were noted to the ventral respiratory column from the MDH, we also suggest a projection to the ventral medullary surface transmitted along gap junctions to somatostatin neurons in the preBötzinger complex may reinforce the apnea (Figure 12; blue line) by disrupting normal rhythm generation. Our data suggest the bradycardia (Figure 12; red line) and peripheral vasoconstriction (Figure 12; orange line) are mediated by neurons in the rostral medulla, and their input by trigeminal neurons is either direct via primary afferent fibers or indirect via the MDH. Since there are inferences implicating that some marine species “will” the DR as well as numerous instances when humans breath-hold, suprabulbar control must intercede in the reflex circuitry, much like when humans induce a response similar to the blink reflex and “wink.”

The universal inclusion of the DR in a wide variety of vertebrates, both marine and terrestrial, is made throughout this review. Indeed, even early diverged mammals, like the platypus, exhibit a dive response (Johansen et al., 1966; Bethge et al., 2003). Although it is utilized best and most by marine mammals, the dive response is also pronounced in non-marine species like common laboratory rodents. While marine mammals have both harnessed the DR as well as adapted numerous systems to prolong underwater submersion, there is no explanation as to why terrestrial animals also have this profound response. It must be remembered, however, that pinnipeds and cetaceans evolved *after* a migration of terrestrial ungulates adapted to an aquatic environment (Thewissen et al., 2007). Thus, the physiological consequences of underwater submersion that we term the DR may have been directed by NSs before marine mammals even existed. This implies that perhaps the moniker “DR” is misleading and in fact a misnomer. Perhaps a purpose of this enigmatic reflex is to indeed to preserve life of the organism (Panneton, 2013).

The neural circuits driving the DR are probably intrinsic in all vertebrate species, implying these circuits are the simplest, the most organized and the most automatic. Those circuits driving the DR also probably were born early in our evolutionary history. The commonality speaks to ancient evolutionary adaptations shared by all vertebrates in their battles against asphyxiation (Hagen, 2018). There are some references in the diving literature about the evolutionary significance of the DR (Hochachka, 1997; Mangum and Hochachka, 1998; Mottishaw et al., 1999), but most of such references are implied only for marine mammals. More work must still be done in dissecting the components of the neural circuits important for the DR, but perhaps more discussions on the teleology of this phenomenal response also are in order to better understand it. The phrase “Master switch of life” (Scholander, 1963), or the striking redistribution of blood supply to organs most essential to life (the heart and brain), may provide new discussion on this phenomenal response.

This essay deals with the neural circuits driving the DR in the medulla of two species of rodents, which are the preferred animals for many laboratory experiments. However, the use of terrestrial rodents to study the mammalian DR does not discount the plethora of data accumulated over the past eighty years, and still being produced, on the DR of marine mammals. As mentioned previously, these large marine mammals are thought to possess considerable intelligence, and, in least in our opinion, make it ethically unfathomable to sacrifice these animals to study their brains. We thus offer these rodent models to those interested in studying the neural control of the mammalian DR. If the profound autonomic changes seen in the DR can be utilized in its clinical implications for SIDS, SUDEP, arrhythmias, stroke, headache, anxiety, and others in humans, perhaps the power of this response may be harnessed for the betterment of mankind. The DR is more than an invariant hardwired response and has many overlying factors effecting the DR are best seen in the variables regulating the HR in marine mammals. We suspect that its neural control in higher marine mammals makes adjustments for many conditions, even those for “anticipated” physiological needs, but eventually the output will traverse the basic circuit described.

Perhaps humans can be trained to harness the incredible power the DR has over the automatic systems which drive our organism, thus mimicking those seen in pinnipeds and cetaceans. The innumerable choices inflicted on contemporary man, from all strata of all societies, parallels that of the rise in anxiety and stress levels; some in the general public already promote inducing the DR as a relaxation technique. The DR’s power over cerebral blood flow may be a fast and efficient way to treat incipient migraine headaches as well as minimize the effects of transient ischemic attacks (TIA) and reduce the ischemic penumbra of stroke. Controlling the powerful DR may open doors into therapies for numerous human pathologies.

## Author Contributions

WP designed the study and wrote the first draft of the manuscript. QG performed the statistical analysis and made comments on the manuscript.

## Conflict of Interest

The authors declare that the research was conducted in the absence of any commercial or financial relationships that could be construed as a potential conflict of interest.

## Supplementary Material

VIDEO S1 A low resolution video showing a trained rat voluntarily diving through an underwater maze. We gave the rats no obvious reward for their endeavors; perhaps their ‘freedom’ (we allowed them to run uncaged on top of a table top) was an inducement. Rats were trained 5 days/week prior to the experiment. See Panneton et al. (2014) and McCulloch (2014), for more details.
